# Differentiation of CD117^+^ Amniotic Fluid Stem Cells towards Nephron Progenitors

**DOI:** 10.12669/pjms.38.6.4887

**Published:** 2022

**Authors:** Hina Jabeen, Mohsin Wahid, Jahan Ara Ain Uddin, Farrukh Mustafa

**Affiliations:** 1 Hina Jabeen Dow Research Institute of Biotechnology & Biomedical Sciences, & Department of Anatomy, Dr Ishrat ul Ebad Khan Institute of Oral Health Sciences, Dow University of Health Sciences, Karachi, Pakistan; 2 Mohsin Wahid Dow Research Institute of Biotechnology & Biomedical Sciences & Department of Pathology, Dow International Medical College, Dow University of Health Sciences, Karachi, Pakistan; 3 Jahan Ara Ain Uddin Department of Gynecology & Obstetrics, Dow University Hospital, Dow University of Health Sciences, Karachi, Pakistan; 4 Farrukh Mustafa Department of Anatomy, Dow University of Health Sciences, Karachi, Pakistan

**Keywords:** Amniotic fluid, Amniotic fluid stem cells (AFSCs), Nephron progenitors cells (NPCs), Regenerative medicine

## Abstract

**Objective:**

The study aimed at isolation of CD117^+^ stem cells from amniotic fluid samples followed by their invitro differentiation towards nephron progenitors that can be potentially used for regenerative medicine studies and to further understand pathways involved in renal pathogenesis

**Methods:**

This experimental study was conducted at Dow Research Institute of Biotechnology and Biomedical Sciences (DRIBBS), Dow University of Health Sciences, OJHA Campus Karachi from November 2019 to December 2020. After patient consent, a Pfannenstiel incision was performed by the gynecologist through abdominal and uterine muscles without cutting into Amniotic Membrane. Using a needle of 5CC syringe connected to sterile Redivac bottle, a blunt end insertion was passed through the membrane and the amniotic fluid was aseptically sucked into Redivac bottle, the ice bag was used for transporting amniotic fluid from hospital to the lab and samples were processed within 60 minutes after collection. Amniotic fluid was centrifuged at 4^º^ C for 20 minutes at 1400xg. After centrifugation the cell pellet was treated for analysis of CD117^+^ cells using flowcytometry, once small percentage of CD117^+^ cells were identified the cells were prepared for differentiation and that was carried out using specific growth factors including BMP4, BMP7, FGF2, and retinoic acid, providing the niche to the stem cells for differentiation towards nephron progenitors which was confirmed by protein expression of Wilms Tumor-1 (WT1) using immunofluorescence analysis. The sample size for this invitro work was n=3.

**Results:**

We successfully isolated small percentage of CD117^+^ cells in amniotic fluid followed by in vitro expansion and differentiation towards nephron progenitor cells (NPCs) using well defined media and growth factors, initially differentiated cells were spindle shaped and showed fibroblastic appearance later at stage of nephron progenitors it attained the shape of rounded big clusters, differentiated cells stained positive for WT1 and negative for cluster of differentiation (CD117). Therefore, confirming the successful isolation and differentiation of amniotic fluid stem cells towards nephron progenitors.

**Conclusion:**

To the best of our knowledge this is the first study from the country on the use of Amniotic fluid stem cells and their differentiation towards nephron progenitors that can be used as substitution source of cell therapy for exploration of renal diseases at cellular and molecular level and potential regenerative medicine applications.

## INTRODUCTION

Amniotic fluid seems to appear at 2^nd^ week of pregnancy and it is a worthy component that hold the baby within the womb of mother.^1^ At about 12 days of gestation a sac is formed within the uterus known as amniotic sac, and throughout pregnancy this sac rests the baby as well as the amniotic fluid. It acts as a defensive fluid for the growing fetus; it supplies mechanical support along with nutrients during embryogenesis, the amniotic fluid act as a cellular reservoir throughout pregnancy. In addition to the possible clinical efficacy of the amniotic fluid, its related concern with isolations are minimum.^2^ Amniotic fluid can be withdrawn securely through second trimester routine amniocentesis (at 15 – 21 weeks of pregnancy), third trimester amino reduction (at 28 weeks or later) or caesarean section (end of gestation). After C-section amniotic fluid along with other gestational tissues like placental membrane, placenta are usually discarded and these tissues are major source of stem cells containing highly multipotent activities.^3^

CD117 positive cells or Amniotic fluid stem cells (AFSCs) are those cells which are derived from amniotic fluid these cells are broadly multipotent that are present in amniotic fluid and can differentiate into various lineages including osteogenic, myogenic, nephrogeneic, neurogenic, epithelial and hepatic cells and these AFSCs can proliferate more rapidly than the surrounding cells and might be constantly isolated from amniotic fluid making it a reliable source of stem cells.^4^

In regenerative medicine a recent effort is focused at isolation of human stem cells that are highly proliferative and easy to collect with substantial plasticity and without ethical issues. AFSCs have all these characteristics.^5^ Looking at the morphological features of amniotic fluid cells, they can be classified into three types, Amniotic fluid (AF-type cells), Epitheloid (E-type cells) and Fibroblastic (F-type cells). AF-type cells are derived from fetal trophoblastic membranes while E-type cells are supposed to come from fetal urine and skin and F-type cells originate from dermal fibroblast and connective tissue.^6^

In adding to pluripotency markers, Early pregnancy AFSCs show high levels of some Mesenchymal stem cells (MSC) markers including CD29, CD73, CD105, CD90, CD44, Human Leukocyte Antigen (HLA) Class-I and Class-II is low or negative, Second and third trimester/full term human AFSCs (Isolated with the “attachment to plastic /CD117 selection “protocol) express c-Myc and Oct-4 but do not express Nanog and Klf4. They also express SSEA4+, but SSEA3+, TRA-1-60, TRA-1-81 and ALP are absent.^7^

### Rationale of the Study

Renal diseases are evolving as a critical problem worldwide and because of limited options for treatment of damaged kidneys; the search for a new therapeutic route is a valuable method for improvement in renal diseases. Therefore, an invitro model to study nephrogenesis and provision of therapeutic cells in acute kidney injury is a need of the time. Therefore, this study aimed at differentiation of amniotic fluid derived stem cells towards nephron progenitors in an invitro system.

Our objective was Isolation of CD117^+^ cells from amniotic fluid and their differentiation towards nephron progenitor cells followed by molecular characterization of isolated and differentiated cell types using flow cytometry and immunofluorescence, respectively.

## METHODS

This experimental study was conducted at DRIBBS (Dow Research Institute of Biotechnology and Biomedical Sciences), Dow University of Health Sciences, Karachi Pakistan. The study duration was from November 2019 to December 2020.

### Inclusion criteri

Pregnant women age less than 35 years, having uncomplicated pregnancies were included in this study. Informed consent was attained from women who underwent elective C-section.

### Exclusion criteria

Pregnant women age more than 35 years, having risk to develop complications. Those having history of Diabetes and hypertension were not included in this study.

### Ethical Consideration

The research project was approved by IRB/Ethics Committee of Dow University of Health sciences (DUHS) (IRB-l084/DUHS/Approval/2018/).

### Amniotic fluid collection

Samples of amniotic fluid were collected by staff of Gynecological and Obstetrical department at Ojha campus Dow University of Health Sciences. A fenistil incision was performed by Gynecologist up to the uterine muscle without cutting the amnion membrane, using a needle connected with Redivac bottle, a blunt insertion was made into amnion layer and amniotic fluid suctioned aseptically into Redivac bottle. The bottle was labeled and the sample was processed with an hour of collection.

### Processing

The Redivac bottle with amniotic fluid was placed aseptically in biological safety cabinet Type-II A2 (Thermo fisher scientific) and amniotic fluid were transferred from Redivac bottle into centrifuge tubes, the tubes were centrifuged (Eppendorff, centrifuge 5810 R) at 4°C for 15 minutes at 1400xg, after centrifugation the supernatant was removed and remaining cell pellet was cultured and characterized as defined below.

### Isolation and Characterization of CD117^+^ (AFSCs) by Flowcytometry

The freshly isolated amniotic fluid cell pellet was resuspended in 3ml Dulbecco Phosphate Buffered Saline (DPBS, Thermo fisher scientific) and then transferred into Fluorescence activated cell sorting (FACS) tubes. For immunophenotyping using Flourochrome conjugated labeled antibodies against cell surface antigen were added one was CD117 as positive and other was cluster of Differentiation (CD34) used as negative then cells were incubated in 4°C for 30 minutes then the cells were examined by using (BD FACS Celesta, Software FACS Diva version 8).

### Differentiation of CD117^+^ stem cells towards NPCs

We differentiated amniotic fluid stem cells (AFSCs) into nephron progenitors (NPCs) following different stages. Initially it was AFSCs, second one primeval streak (PS) third was Intermediate mesoderm (IM) then finally NPCs via using various growth factors respectively. First undifferentiated AFSCs were replated on Geltrex (1:100 dilution Gibco # A1413201) coated six well plate and cultured in mTeSR1 basal medium (stem cell technologies #AD11892266) supplemented with 10ng/ml Fibroblast growth factor two (FGF2) for four days in feeder free method then AFSCs were switched to basal differentiation medium Rose well park memorial institute (RPMI) medium containing 2% B27, 1% pen/strep) supplemented with 100ng/ml Wingless related integration site (Wnt3a) for one day and then following two days AFSCs were supplemented with Bone morphogenic protein 4 (BMP4) 20ng/ml (PeproTech Biocompare) along with 10ng/ml FGF2, for differentiate into intermediate mesoderm BMP4 was replaced with Bone morphogenic protein 7 (BMP7) 50ng/ml (PeproTech Biocompare) in similar RPMI medium treated with 10ng/ml Retinoic Acid (Alfa aesar A044540.77) and 10ng/ml FGF2 for four days, lastly in final step of NPCs were treated with BMP7 150ng/ml and FGF2 50ng/ml which was designed for 14 days.

### Immunocytochemistry

The amniotic fluid samples were washed thoroughly with Phosphate Buffered Saline then fixed at 1:1 acetone: methanol for twenty minutes at 25°C, after washing with Buffer Saline containing tween 20 (PBST) three times Triton X-100 was use for permeabilization of cell membrane then again washed with PBST, it was clogged by bovine serum albumin (BSA) 1% plus PBS (without Calcium &Magnesium) for 30 minutes at Room Temperature and incubated with primary monoclonal unconjugated antibody WT1 (WT1/857 + 6F-H2 clone, Novus Biologicals, Biotechne, USA) as positive and CD117 (CD117 APC, Thermofisher, 104D2) as a negative control for an hour. After washing thrice with PBS the samples were incubated with secondary antibodies, Goat Anti-human IgG – Fluorescence isothiocynate (FITC) and Goat Anti-Mouse IgM- Texas Red for an hour, after rinsing three times with PBS the samples were fixed with (Diaminodino-2- phenylindole) DAPI for ten minutes and stain cells were observed by using Dmi8 Fluorescence microscope (Leica microsystem).

## RESULTS

**Fig.1:**
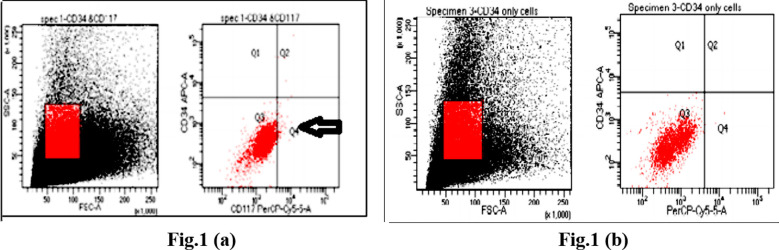
Analysis of AFSCs via using flowcytometry, within two independent preparations of AFSCs the presence of (a) CD117 positive cells was less than 1% as shown in Q4 indicated by arrow and (b) devoid of CD34 hematopoietic marker.

Flowcytometry analysis of cell surface marker present in amniotic fluid, specific cell surface antigen CD117^+^ and CD34 negative marker were analyzed by flow Cytometry, 1.5 million cells from amniotic fluid were washed and resuspended in phosphate buffer saline and transferred into FACS tubes and cells were stained with both antibodies for cell surface markers including CD117 (Fluorochrome PerCp-Cy 5.5) and CD34 (Fluorochrome APC) but having different fluorochrome. Analysis was carried out using BD FACS Celest., Fig.1 Amniotic fluid expressed approximately 1% of CD117^+^ andCD34 expressed negative. This was performed on three different biological samples n=3.

**Fig.2:**
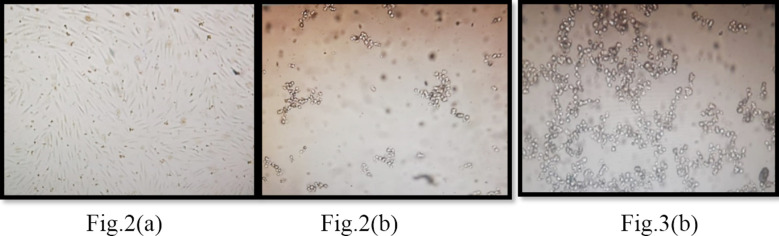
Morphological changes of AFSCs at every stage (A) Day 0- 4 cells changed their morphology and become spindle shape fibroblast (B) Day 7-11 cells were appeared as small rounded cluster and finally on (C) Day 11-24 at NPCs stage cells were seen a big rounded clusters under phase contrast microscope at 10X magnification.

Cells were not sorted and placed directly in to cell culture plates after confirmation from flowcytometry that although in very small percentage but still CD117^+^ cells were present in the amniotic fluid samples. In order to create optimal condition for differentiation, we used 24 days protocol by treating with two different media in combination with growth factors. After centrifugation of amniotic fluid at 1400xg at 4°C for 20 minutes, the supernatant was removed and remaining cell pellet was expanded and amplified in mTeSR medium along with FGF2 for four days after that the cells gives appearance of spindle shape as shown in Fig.2 (a) then medium switched to RPMI and supplemented with FGF2 and BMP4 for two to three days and then treat with Wnt3a for one day, for subsequent four days cells were cultured with BMP7, FGF2 and Retinoic acid at this stage cells become change their shape convert in rounded small clusters as shown in

Cells were treated in basal medium but augmented with high dose Bone morphogenic protein7 (BMP7) 150ng/ml along with 50ng/ml FGF2 without Retinoic acid for 14 days and at this stage of NPCs, cells morphological gives features of rounded big clusters as shown in Fig.2(c).

**Fig.3A:**
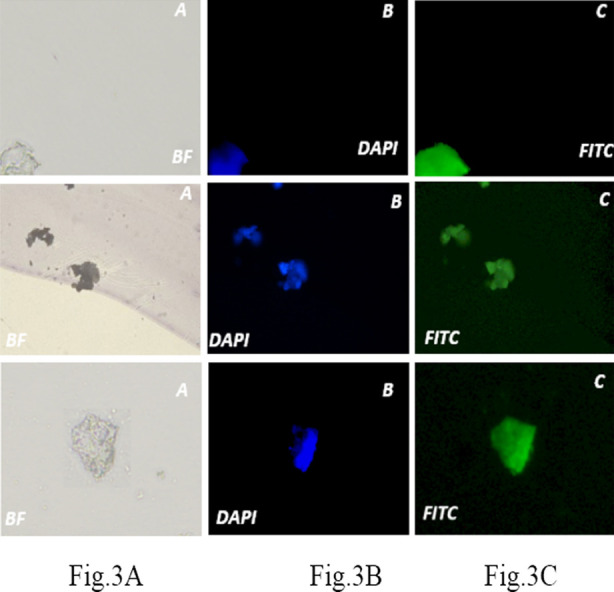
Characterization of NPCs colonies for WT1 by immunofluorescence staining as performed on three different amniotic fluid derived NPCs. This figure shows immunofluorescence analysis for positive nuclear marker WT1 (a) Bright field image of NPCs at 40x showed irregular with ill-defined borders having tightly packed cells for the three different amniotic fluid derived NPCs. (b) Counter DAPI nuclear stain showed blue color of Nucleus. (c) Characteristic of NPCs, WT1 is shown in nuclear compartment of cells.

In this study, we confirmed the presence of positive nephron progenitor cells marker WT1, and absence of negative marker CD117. WT1 is nuclear marker which is encoded by WT1 gene responsible for cell proliferation and survival of NPCs, it plays a relevant role in induced renal progenitors during mesengial epithelial transition^11^, on the other hand CD117 (c kit) is tyrosine kinase receptor it is not present in any differentiation stage of nephron progenitor cells, it is used as negative marker. Immunostaining of nephron progenitor cells with WT1 showed expression as shown in Fig.3 A Cell nucleus were stained with DAPI. Immunostaining of NPCs with CD117 showed no protein expression for the marker confirming that CD117 stem cells have now been differentiated towards nephron progenitors which do not express CD117.

## DISCUSSION

As compared to other organs in our body, kidney is made up of more specialized cells called as functional units; therefore formation of nephron progenitors is more suitable approach to regenerate injured cells within functional units because after administration of progenitors into damaged tissues, progeny cells have potential to make any type of cell in human body. AFSCs have the ability to differentiate into all three germ layers,^8^ progressively these cells are involved in tissue regeneration including kidney,^9^ lungs, bone and bladder.^10^

In this study, we isolated and characterized the CD117^+^ cells (AFSCs) from Amniotic fluid, the amniotic fluid were collected during C-section, which were usually discarded at time of birth.^11^ A comparison with other studies showed that CD117^+^ cells can be isolated from 2^nd^ trimester also and isolation of CD117^+^ cells from amniotic fluid (less than 1% of total amniotic fluid cells) is also carried various protocols as like via magnetic cell sorting or flow cytometry.^12^

Amniotic fluid contain heterogeneous population of cells but among all AFSCs have both recompenses over adult and embryonic stem cells like pluripotent action, immunomodulatory properties and high plasticity that can be a rich source off future therapeutic uses.^13^

In this study by using flowcytometry technique it has been shown that less then 1% population of Amniotic fluid stem cells (CD117^+^ cells) were present in the samples which were characterized by using antibodies against cell surface marker, for further conformation we also used negative marker CD34, which were not detected in amniotic fluid.^14^

At stage of nephron progenitor cells it is very important to express the protein at molecular level, which we confirmed using Immunofluorescence, the one positive marker (WT1).^15^ One negative marker (CD117), and secondary antibodies used as isotype control. In the light of afore mentioned studies, it is recommended that amniotic fluid stem cells potentially be used in vivo as well as vitro studies 16. AFSCs have the inherent capability to differentiate into various cell types that can develop into the renal tissue and can also represent a potential, natural source of cells for renal tissue engineering. Methods involving stem cells to treat diseases reduce the symptoms of the disease to a great extent which eventually helps patients to be less dependent on medicines.

Amniotic fluid stem cells have been isolated and differentiated towards nephron progenitors. We confirmed the nephron progenitor cells by using WT1 antibody which is strongly expressed in NPCs, while previous studies it was observed that along with WT1, the expression of Homeobox protein-2 (SIX2) also validate at stage of nephron progenitors. The study has provided a source of stem cells which is usually discarded and confirmed the small population of amniotic fluid stem cells that are available for regenerative medicine purposes. The differentiation towards NPCs is very important to facilitate the applications for tissue engineering, disease modeling and drug screening applicable in acute kidney injury in terms of a therapeutic option.

### Limitations of the study

The molecular characterization of nephron progenitor cells was shown by protein expression of WT1 marker which is specific for these cells; however, more markers could be used for this purpose but couldn’t be done due to budget constraints. 

## CONCLUSION

To the best of our knowledge this is the first study from the country on the use of Amniotic fluid stem cells and their differentiation towards nephron progenitors that can be used as substitution source of cell therapy for exploration of renal diseases at cellular and molecular level and potential regenerative medicine applications.

### Authors’ Contributions:

**MW:** Designed the study and supervised all the research work involving Stem cells isolation, differentiation and expansion, multicolor flowcytometry and Immunofluorescence and final approval of the manuscript. MW is responsible for the integrity and accuracy of the work.

**HJ:** Performed all the experiments under supervision and prepared the Manuscript.

**JA:** Provided the technical support and facilitated amniotic fluid collection and critical review for the manuscript.

**FM:** Provided technical support and critically reviewed the manuscript.
